# Modelled air pollution levels versus EC air quality legislation - results from high resolution simulation

**DOI:** 10.1186/2193-1801-2-78

**Published:** 2013-03-05

**Authors:** Hristo Chervenkov

**Affiliations:** National Institute of Meteorology and Hydrology – branch Plovdiv, Bulgarian Academy of Sciences, Plovdiv, Bulgaria

**Keywords:** Air pollution simulation, Long-term air pollution levels, EC-directives, Pollutant exceedances

## Abstract

An appropriate method for evaluating the air quality of a certain area is to contrast the actual air pollution levels to the critical ones, prescribed in the legislative standards. The application of numerical simulation models for assessing the real air quality status is allowed by the legislation of the European Community (EC). This approach is preferable, especially when the area of interest is relatively big and/or the network of measurement stations is sparse, and the available observational data are scarce, respectively. Such method is very efficient for similar assessment studies due to continuous spatio-temporal coverage of the obtained results. In the study the values of the concentration of the harmful substances sulphur dioxide, (SO_2_), nitrogen dioxide (NO_2_), particulate matter - coarse (PM_10_) and fine (PM_2.5_) fraction, ozone (O_3_), carbon monoxide (CO) and ammonia (NH_3_) in the surface layer obtained from modelling simulations with resolution 10 km on hourly bases are taken to calculate the necessary statistical quantities which are used for comparison with the corresponding critical levels, prescribed in the EC directives. For part of them (PM_2.5_, CO and NH_3_) this is done for first time with such resolution. The computational grid covers Bulgaria entirely and some surrounding territories and the calculations are made for every year in the period 1991–2000. The averaged over the whole time slice results can be treated as representative for the air quality situation of the last decade of the former century.

## Introduction

Clean air is essential for a good quality of life and it enhances the social well-being of European citizens. Scientific assessments reveal a range of harmful effects from the past and present levels of air pollution including health problems, respectively premature deaths (Jerrett et al 2005), reduced agricultural crop yields, changes in ecosystem species composition, and damage to physical infrastructure and cultural heritage due to material deterioration etc. There is now common scientific understanding that all the important air quality problems mentioned above are strongly interrelated. All these pollutants are subject to long-range transport and various transformations in the atmosphere, so that concentrations experienced at a given site originate from a large number of diverse emission sources across Europe. Thus, effective strategies for reducing pollution levels cannot be developed solely at a local scale, but need international cooperation. In this sense keeping air pollution limits under certain, prescribed in international legislative norms, critical levels, is an important environmental issue for each country. In the study the values of the concentration of seven harmful substances in the surface layer, namely sulphur dioxide, (SO_2_), nitrogen dioxide (NO_2_), particulate matter - coarse (PM_10_: particulate matter which passes through a size-selective inlet with a 50% efficiency cut-off at 10 μm aerodynamic diameter) and fine (PM_2.5_: same as PM_10_, but for 2.5 μm) fraction, ozone (O_3_), carbon monoxide (CO) and ammonia (NH_3_), obtained from high-resolution modelling simulations on hourly bases, are taken. Very frequently the last two pollutants are not evaluated and this work is a step toward fulfilling this gap. Seventeen statistical quantities are calculated, part of them for first time for this domain with this resolution, which are used for comparison with the corresponding critical levels prescribed in the EC directives. The pollutants, selected for consideration here, form only part of the vast range of air substances that have ecological effects. The computational grid covers Bulgaria entirely and the calculations are made for every year in the period 1991–2000. The averaged results over the whole time slice, presented here, can be treated as representative for the air quality situation of the last decade of the former century.

## Short summary of the air quality legislation in the European community

Many countries of the European Region encounter similar air pollution problems, partly because pollution sources are similar, and in any case air pollution does not respect national frontiers. The subject of the transboundary long-range transport of air pollution has received increasing attention in Europe over the last decades. In the mid-1990s, in the context of the Fifth Environmental Action Programme, the European union (EU) launched a major reform of its air quality (AQ) legislation aimed at providing more effective protection of people against health risks from air pollution and at better protection of the environment, including the objective of ensuring that critical loads and levels for acidification in the Community were not to be exceeded. This effort began with the adoption of the 1996 Air Quality Framework Directive (AQFD), which establishes a common strategy for

1. defining ambient AQ objectives to protect human health and the environment;
2. assessing ambient AQ on the basis of uniform methods and criteria;
3. making information on AQ available to the public, including by means of alert thresholds; and
4. “maintain[ing] where it is good and improve[ing] it in other cases”

The actual setting of air quality limit values (AQLVs) and alert thresholds for specific pollutants is done via the so-called Daughter Directives, namely

-  Council Directive 1999/30/EC relating to limit values for sulphur dioxide, nitrogen dioxide and oxides of nitrogen, particulate matter and lead in ambient air (First Daughter Directive).
-  Directive 2000/69/EC of the European Parliament and of the Council relating to limit values for benzene and carbon monoxide in ambient air (Second Daughter Directive).
-  Directive 2002/3/EC of the European Parliament and of the Council relating to ozone in ambient air (Third Daughter Directive).
-  Directive 2004/107/EC of the European Parliament and of the Council relating to arsenic, cadmium, mercury, nickel and polycyclic aromatic hydrocarbons in ambient air (Fourth Daughter Directive).

The AQLVs are defined as hourly, daily or annual/seasonal arithmetic averaged concentration thresholds which should not be exceeded on more than a given number of occasions. They represent long-term objectives equivalent to the World Health Organisation’s (WHO) new guideline values and are summarized in Table 1. According First Daughter Directive, stage 1 is the already entered into force legislation, and stage 2 means “Indicative limit values to be reviewed in the light of further information on health and environmental effects, technical feasibility and experience in the application of Stage 1 limit values in the Member States”. Because the ammonia is out of the scope of the above mentioned legislative documents, the WHO-guideline values (Chan M., Danzon M., (eds) 2006) are used for evaluation of the pollution with this substance. In general, the guidelines address single pollutants, whereas in real life exposure to mixtures of chemicals occurs, with additive, synergistic or antagonistic effects. In dealing with practical situations or standard-setting procedures, therefore, consideration should be given to the interrelationships between the various air pollutants. Because these new values are considerably lower than the previous AQLVs, and therefore require major pollution reduction efforts in order to achieve attainment, temporary margins of tolerance are set for certain pollutants. These margins of tolerance are then reduced stepwise, so as to provide interim targets until the AQLV is attained at the end of the determined period. Besides setting numerical AQLVs and alert thresholds (in the case of ozone, target values) for each of the identified pollutants, the Daughter Directives harmonise monitoring strategies, measuring methods, calibration and quality assessment methods to arrive at comparable measurements throughout the EU and to provide for good public information.

**Table 1:** **Synthesis table of the EU-legislation and WHO-proposed AQLV (concentration thresholds (unit: μg/m**^**3**^**)/maximum allowed number of exceedances) under consideration**

Av.period/Parameter Pollutant	1 hour	Maximum daily 8-hour mean	24 hours	Winter (1 October-31 March)	Calendar year
SO_2_	350/24		125/3	20/0	20/0
NO_2_	200/18		75/0^1^		40/0^2^ 30/0^3^
O_3_		120/25^4^ 120/0^5^			
PM_10_			50/35^6^ 50/7^7^		40/0^6^ 20/0^7^
PM_2.5_					24/0
CO		10000/0			
NH_3_			270/0^1^		8/0^1^

1) WHO-proposed AQLV.

2) Annual limit value for the protection of human health.

3) Annual limit value for the protection of vegetation (for NO_x_).

4) Target value.

5) Long-term objective.

6) On stage 1.

7) On stage 2.

In year 2008 the AQFD was repealed with new directive (Directive 2008/50/EC) on ambient air quality and cleaner air for Europe including the following elements:

-  The merging of most of the existing legislation into a single directive (except for the Fourth Daughter Directive) with no change to existing air quality objectives.
-  New air quality objectives for PM_2.5_ (fine particles) including the limit value and exposure related objectives – exposure concentration obligation and exposure reduction target.
-  The possibility to discount natural sources of pollution when assessing compliance against limit values.
-  The possibility for time extensions of three years (PM_10_) or up to five years (NO_2_, benzene) for complying with limit values, based on conditions and the assessment by the European Commission.

The circumstance that this legislation was not completely effective in the investigating decade is not significant, because the aim of the study to evaluate the air pollution status in Bulgaria from the viewpoint of the contemporary knowledge, is on the principle of the above-mentioned directives.

## Concept and methodology

Chemical Transport Models (CTM) are an essential computational method used to predict air quality, which are closely aligned to weather prediction models. They also incorporate the complex reactions of pollution emissions, their dispersal in the atmosphere, the chemical transformations they undergo and their removal processes. Contemporary CTM solely or combined with other modern data sampling and processing facilities are the computational core of broad range of European initiatives such as GMES (Europe’s initiative for Global Monitoring of Environment and Security), HARMO (Harmonisation within Atmospheric Dispersion Modelling for Regulatory Purposes), ACCENT (Atmospheric Composition Change, the European Network) and others. GMES is the name of the Earth Observation programme launched by the European Commission and the European Space Agency. The objective of GMES is to monitor and forecast the state of the environment on land, at sea and in the atmosphere and to improve the security of the citizens in a world facing an increased risk of natural and other disasters. GMES does not replace existing European capacities, but rather complements them with a view to fulfilling user needs and guaranteeing sustainability and European Earth Observation autonomy in the long term. MACC-II - Monitoring Atmospheric Composition and Climate - Interim Implementation (http://www.gmes-atmosphere.eu/services/raq) - is the current pre-operational GMES Atmosphere Service. MACC-II provides data records on atmospheric composition for recent years, data for monitoring present conditions and forecasts of the distribution of key constituents for a few days ahead. MACC-II combines state-of-the-art atmospheric modelling with Earth observation data to provide information services covering European air quality, global atmospheric composition, climate forcing, the ozone layer and UV and solar energy, and emissions and surface fluxes. The principal goal of other initiative, the PASODOBLE project, (http://www.myair-eu.org) is to develop and demonstrate user-driven information services (MYAIR services) for the regional and local air quality sector by combining space based data, in-situ data and models in four thematic service lines. Through this project European citizens will directly benefit from earth observation, measurement networks and air quality modelling. The project infrastructure includes interfaces to GMES Core Services – satellite based measurements and in-situ data.

The present work is based on the primary results obtained in a modelling study preformed in the Bulgarian National Institute of Meteorology and Hydrology (NIMH), in the frame of the EC FP6 Project “Central and Eastern Europe Climate Change Impact and Vulnerability Assessment” (CECILIA) program. CECILIA’s primary mission was to improve the understanding of local climate change in Central and Eastern Europe and its impacts on forestry, agriculture, hydrology and air quality. The main objective of the project was to deliver a climate change impact and vulnerability assessment in targeted areas of Central and Eastern Europe. Emphasis is given to applications of regional climate modelling studies at a resolution of 10 km for local impact studies in key sectors of the region Juda-Rezler et al (2012). Specific tasks of the activities in CECILIA’s workpackage 7 (WP7) was to study the impacts of climate change on health and air quality (photochemistry of air pollution, aerosols). For this purpose special modellig system (Syrakov et al 2010; Syrakov et al 2011) was build in NIMH - BAS Sofia, which computational core is the US EPA Models-3 (Byun and Ching 1999 , Byun and Schere 2006) air quality modelling, consisting of:

-  CMAQ - Community Multi-scale Air Quality model being the CTM of the Models-3 System;
-  MM5 - the 5th generation PSU/NCAR Meso-meteorological Model used as meteorological pre-processor to CMAQ, and
-  SMOKE - Sparse Matrix Operator Kernel Emissions Modelling System being the emission pre-processor to CMAQ.

A number of interfaces (Linux scripts and FORTRAN codes) are created as to link those models with different types input information in a system able to perform long term calculations. Meteorological driver of the system is the ALADIN weather forecasting model Spiridonov et al. (2005) operating in this case in special mode. In spite MM5 performs full meteorological modelling, here it is used as a kind of dynamical pre-processor in space and time. The domain’s vertical profile contained 23 σ-levels of varying thickness, extending up to 100 hPa height. Proper physical options are set in MM5. The speciation procedure is dependent on the Chemical Mechanism (CM) used. CMAQ supports different CMs. Here, the Carbon Bond, v.4 (CB4) is exploited. The basis of the CB-4 mechanism is that reactivity of organic compounds in the atmosphere can reasonably be simulated by mechanism species that represent different carbon bond types. In the time passing CB4 has undergone several changes since its publication. In particular particle mater chemistry is added. In the used Version 4.6 of CMAQ the CB4 is upgraded with the Version 1.7 of ISORROPIA aerosol model Nenes et al. (1998). According to this combined mechanism (CB4-aero3) 10 organic and 5 PM_2.5_ spices are input in addition to the other inorganic gases. Most of the organic species in CB4 represent carbon-carbon bond types, but ethene (ETH), isoprene (ISOP) and formaldehyde (FORM) are represented explicitly. A new method for computing the vertical velocity has been implemented which follows the omega calculation in the Weather Research and Forecasting (WRF) model but using CMAQ’s advection schemes to compute horizontal mass divergence. It’s a two step process where first the continuity equation is integrated through the column to get the change in column mass and then solve for omega layer-by-layer using the horizontal mass divergence. The new scheme is much less diffusive in the upper layers because it is constrained to have zero flux at the model top. The dry deposition is modeled using the electrical resistance method. The wet removal processes can proceed first with the cloud droplet formation via several mechanisms including heterogeneous nucleation and aerosol activation, then with in-cloud scavenging by existing cloud droplets or below-cloud scavenging by falling precipitation or both. All of these processes influence the amount and composition of the ground-level rainwater. Therefore, an accurate parameterization scheme, describing all these processes is incorporated in the system.

As shown in Chervenkov et al. (2008), the long range transport of pollutants can play relevant role even for not long-living pollutants like SO_2_. The modelling system needs adequate boundary conditions (BC). The necessary for the BC condition data are prepared with the Comprehensive Air quality Model with eXtensions (CAMx) Katragkou et al. (2008) by means of off-line procedure that takes place in Aristotle University of Thessaloniki in Greece. The results are uploaded to a dedicated server in Sofia. There, this data is processed online in order to produce the current day CMAQ-ready boundary condition file. Due mainly to the specifics of the coordinate system, the CMAQ do not requires upper boundary conditions.

CMAQ demands its emission input in specific format, reflecting the time evolution of the release of all pollutants accounted for by the used chemical mechanism. The emission inventory is usually made on annual basis for, as a rule, big territories (municipalities, counties, countries, etc.) and many pollutants are estimated as groups like NO_x_, SO_x_, VOC (Volatile Organic Compounds), PM_2.5_ In preparing CMAQ emission file a number of specific estimates must be done. Firstly, all this information must be gridded. Secondly, time variation profiles must be over-posed on these annual values to account for seasonal, weekly and daily variations. Finally, organic gases emission estimates, and to a lesser extent SO_x_, NO_x_ and PM_2.5_, must be split, or ‘speciated’, into more defined compounds in order to be properly modeled for chemical transformations and deposition. The different types of sources: Area Sources (AS), Large Point Sources (LPS), mobile and Biogenic Sources (BS) are treated in specific way. Obviously, emission models are needed as reliable emission pre-processors to the chemical transport models. Such a component in EPA Models-3 system is SMOKE. Unfortunately, it is highly adapted to the US conditions – emission inventory, administrative division, motor fleet etc. Many European scientific groups are working now for adapting SMOKE to European conditions Borge et al (2008). SMOKE is partly used here, only for to calculate biogenic emissions and to merge AS-, LPS- and BS-files into a single CMAQ emission input file. The anthropogenic emission files (AS and LPS) are prepared by interface programs. Input to these interfaces is gridded emission inventories. Here, TNO high resolution inventory Visschedijk et al (2007) is exploited. The TNO inventory resolution is 0.25° × 0.125° longitude-latitude, that is on average 15 × 15 km. GIS technology is applied to transform this data to the CMAQ grid and so to produce the correct input. It must be mentioned that the TNO inventory is elaborated for AS and LPS separately, distributed over 10 SNAPs (Selected Nomenclature for Air Pollution). The temporal allocation is made on the base of daily, weekly and monthly profiles, provided by Peter Builtjes Builtjes et al., (Builtjes et al. 2003). The temporal profiles are country-, pollutant- and SNAP-specific.

SMOKE is used to produce biogenic emission file. The SMOKE system uses a more advanced emissions modelling approach for biogenic processing than it uses for the other source types. For biogenic emissions, the temporal processing is a true simulation model driven by ambient meteorology and other data. SMOKE currently supports BEIS (Biogenic Emissions Inventory System) mechanism, versions 2 and 3 (here version 3.13, see Schwede et al, 2005). BEIS2 and BEIS3 are fed with spatial allocation of land-use data as the first processing step. They subsequently compute normalized emissions for each grid cell and land use category. The final step is adjusting the normalized emissions based on gridded, hourly meteorology data and assigning the chemical species to output a model-ready biogenic emissions file. To isolate the effect of climate change on air quality, anthropogenic emissions scenario for year 2000 was used in all simulations, while climate sensitive biogenic emissions were allowed to vary with the simulated climate. Due to the generally decreasing trend in the anthropogenic share in the emissions in the decade, it is reasonable to expect that this approach underestimates to a certain extend the real air pollution situation. Nevertheless the study is appropriate both because the emission abatement in Bulgaria is not so well expressed as in other European countries. On the other hand, the calculations are performed with very precise emission inventory and high spatio-temporal resolution with modern simulation model system. The achieved results can be treated as estimation of the lower limit of the real Syrakov et al. (2011)) The model grid consists of 54 × 40 grid cells with size 10 × 10 km and covers Bulgaria entirely, together with the border regions of the neighboring countries and the most western part of the Black sea. From the modelling system output only values for the surface layer are stored. Saved on hourly basis are 17 most important pollutants, including all under consideration. The calculated datasets for every day in the decade 1991–2000 are saved in the so-called “Control Run” data base. The needed hourly concentrations for the computation of the statistical quantities, required for the comparison from the directives, are taken from this data base and processed. On the next step the additional condition, namely this for the tolerated number of exceedances, is checked. If, due to any reason (for instance different stages of one directive) for some pollutant we have “duplicate” formulation of the AQLV and the attached additional condition, the more severe one is checked first. This is done in the following manner: If, say, for a given pollutant the directive allows m exceedances of the corresponding statistical average, we find the *(m + 1)*th maximum from all possible values. If this value is greater than the prescribed AQLV, the legislation is breached. This task, namely to find the *m*-th largest among n concentrations has to be repeated many times in each grid cell and that’s why it is essential to optimize the corresponding numerical routine. This is done by setting especially effective procedure by *m < <n*, because this condition is very well expressed. In all cases we find the maximal value also due to its relevance as indicative of the air pollution. All statistical quantities are calculated for every year in the period 1991–2000 and the averaged slice results over the whole time are presented in the next chapter.

## Results

A huge number of numerical studies are nowadays dedicated to the air pollution over Europe. Many of them are performed by a variety of different assumptions, diverse climate and/or pollutant emission scenarios, in particular. According to the reality however, one of the most reliable and comprehensive source of information about this and the other problems linked to them are the EMEP (European Monitoring and Evaluation Programme) summary and status reports (Eliassen et al. (1998 ), Olendrzynsky 1999, Tarrason and Schaug 1999, Tarrason et al 2004, Tarrason et al 2006). In most atmospheric pollutants the emission trend plays a main role in the degree of the air pollution of this substance and its derivatives. In recent decades, major efforts have been made to reduce air pollution in the European Region. The emission of the main air pollutants has declined significantly. For what was probably the most severe air pollutant in many decades in the former century, sulphur dioxide, the effect is most pronounced: its total emission was reduced by about 50% in the period 1980–1995. This abatement, however, is significantly irregularly distributed – in north and west Europe in a relevantly greater degree in comparison with the southeastern part of the continent. The economic collapse in the most eastern countries is the main reason for the emission abatement Chervenkov et al. (2008). In contrast, this tendency in the next decade (2001–2010) is based on consequential environmental policy, in particular the adopting of the EC directives. Some countries, Turkey for instance, report a monotonous increase of emissions for the whole decade **(Tarrason and Schaug**^1999^).

**Figure 1:**
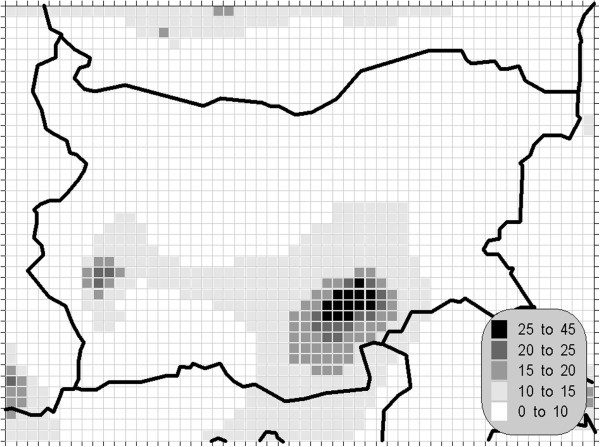
**Annual mean concentration of the sulphur dioxide (unit: μg/m**^**3**^**).**

**Figure 2:**
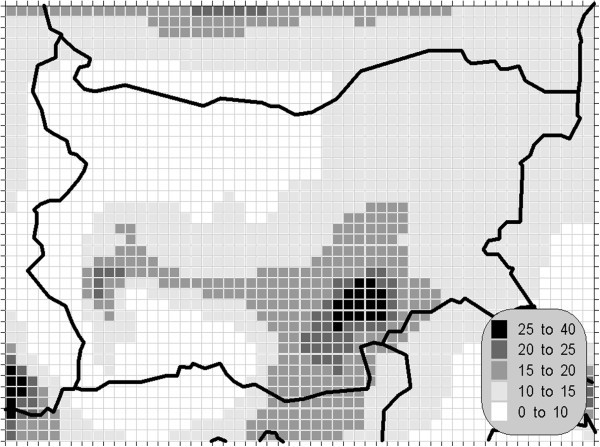
**Winter mean concentration of the sulphur dioxide (unit: μg/m**^**3**^**).**

**Figure 3:**
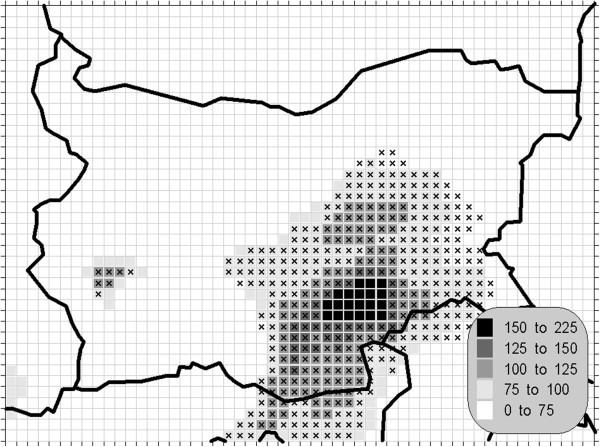
**98.90 percentile (4**^**th**^**daily maximum) of the daily mean concentration of the sulphur dioxide (unit: μg/m**^**3**^**).** The gridcells, where the daily mean AQLV is bridged at least once (1^th^ daily maximum), are marked with crosses.

**Figure 4:**
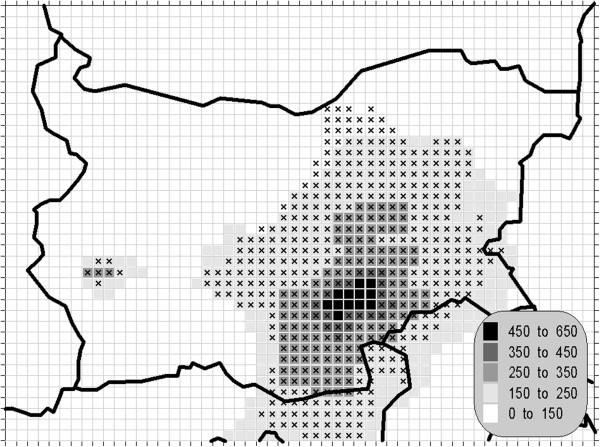
**99.71 percentile (25**^**th**^**hourly maximum) of the hourly concentration of the sulphur dioxide (unit: μg/m**^**3**^**).** The gridcells, where the hourly AQLV is bridged at least once (1^th^ hourly maximum), are marked with crosses.

The atmospheric air pollution with sulphur dioxide is in the base of a variety of processes with deep environmental consequences. The emissions and air concentrations of this substance are in the scope of the earliest air quality legislations not only in Europe. These processes differ significantly in the time scale and this is the main reason for the fact, that in the contemporary directives this pollutant is with the greatest number of defined AQLV. The norms cover a wide range of time spans from hours (in some other documents even 10-minute threshold is prescribed) to a year. The distribution of the calculated statistics is shown on Figures 1, 2, 3 and 4.

**Figure 5:**
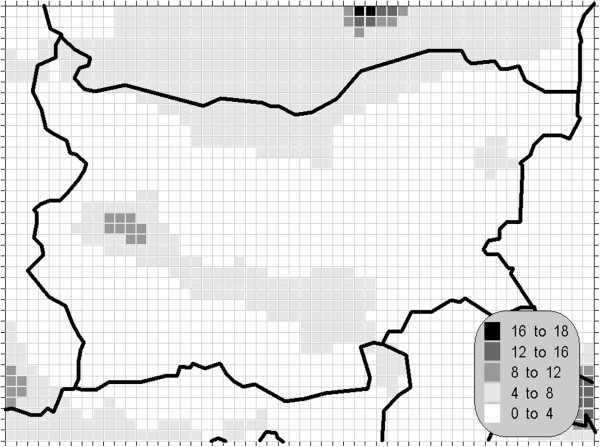
**Annual mean concentration of the oxides of nitrogen (unit: μg/m**^**3**^**).**

**Figure 6:**
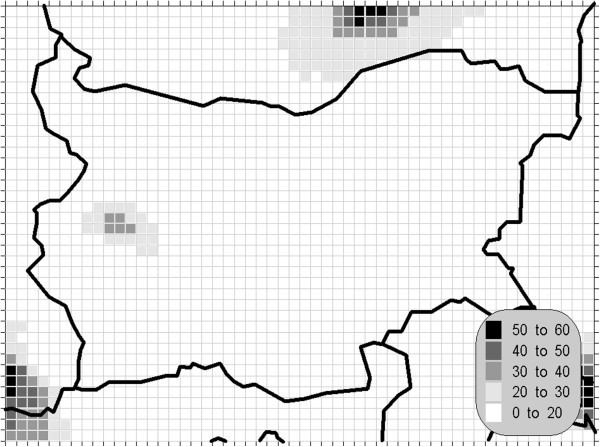
**Maximal daily mean concentration of the nitrogen dioxide (unit: μg/m**^**3**^**).**

**Figure 7:**
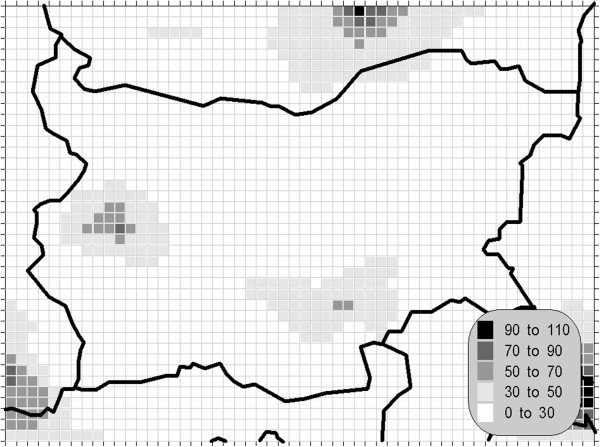
**Maximal hourly concentration of the oxides of nitrogen dioxide (unit: μg/m**^**3**^**).**

In the observed decade, the reduction of emission of nitrogen oxides was smaller in comparison with the sulphur dioxide: the total emission declined by about 15% in the period from 1990 to 1995 Theakston (2002). The nitrogen dioxide, together with the sulphur dioxide and other ozone precursors, are important both because of the high anthropogenic amounts produced and because of their wide distribution. These substances deserve special attention due to significant adverse effects on ecological systems in concentrations far below those known to be harmful to humans. The 24-hour mean has not prescribed AQLV in the above-mentioned document, but due to the environmental significance of this compound we have calculated this statistical measure and compared with the proposed in Theakston (2002) value of 75 μg/m^3^. The obtained results are shown on Figures 5, 6 and 7.

**Figure 8:**
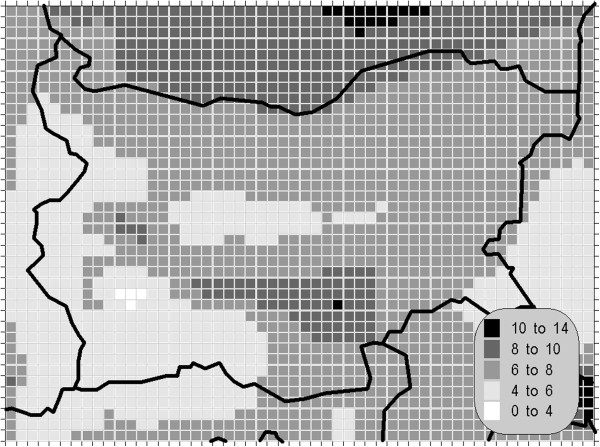
**Annual mean concentration (unit: μg/m**^**3**^**) of the PM**_**10**_**.**

**Figure 9:**
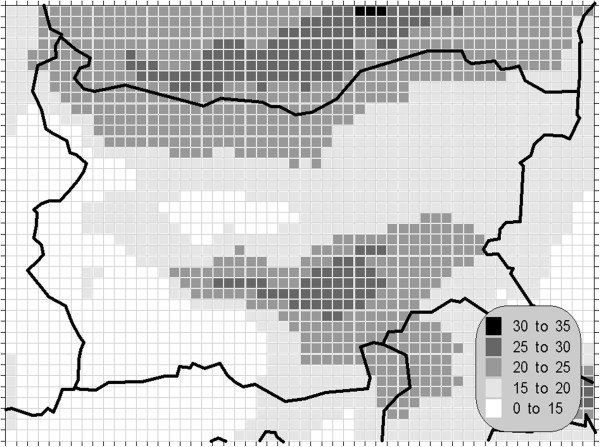
**97.81 percentile (8**^**th**^**daily maximum) of the daily mean concentration of the PM**_**10**_**(unit: μg/m**^**3**^**).**

**Figure 10:**
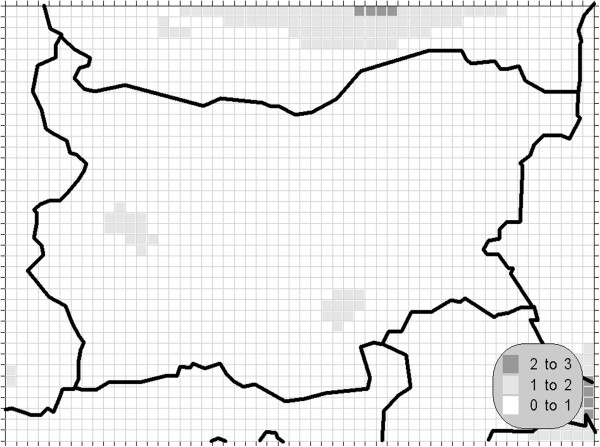
**Annual mean concentration (unit: μg/m**^**3**^**) of the PM**_**2.5.**_

Traditionally, particulate matter air pollution has been thought of as a primarily urban phenomenon. It is now clear that in some areas of Europe the background concentrations are relatively high, indicating that particulate matter exposure is widespread. Similar to the nitrogen oxides, the trend in concentrations in urban air of this compound is less clear in comparison with the sulphur dioxide and it is envisaged that these pollutants still constitute a risk to human health and environment. Limited evidence from studies on dust storms however indicates that PM_10_ particles are much less toxic than those associated with combustion sources, for instance from incinerator plants. Recent studies in which PM_10_ size fractions and/or constituents have been measured suggest that the observed effects of PM_10_ are in fact largely associated with fine particles, strong aerosol acidity or sulfates (which may serve as a proxy for the other two) and not with the coarse (PM_10_ minus PM_2.5_) fraction. Smaller particles that are able to penetrate deeply into the lungs represent a greater risk to health than larger particles (Buonanno et al (2011)). Due to this and other reasons it is clear that the aerosol pollution has to be evaluated separately for these (at least) two size modes in observational and in such numerical studies, whose results are displayed on the Figures 8, 9 and 10.

**Figure 11:**
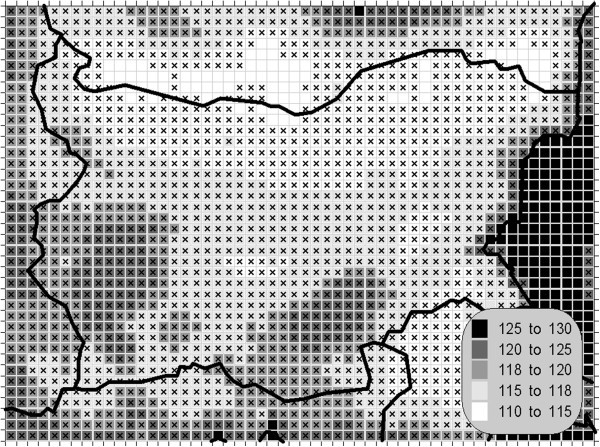
**92.88 percentile (26**^**th**^**maximum of this statistics) of the running 8-hour mean concentration of the ozone (unit: μg/m**^**3**^**).** The gridcells, where the running 8-hour mean AQLV is bridged at least once (1^th^ maximum), are marked with crosses.

It is well-known fact that the ozone differs from the other contaminants under consideration in two significant features. First, it is a secondary pollutant – it is formed in the atmosphere through chemical reactions between nitrogen oxides and volatile organic compounds (VOC) in the presence of short-wavelength radiation from the sun during a timescale from hours to days. Second, the concentration of this pollutant depends to a greater extent, then those of the others, on meteorological conditions, especially the sunlight intensity and the temperature. Due to these reasons the link between (precursor) emissions and concentration is much more complicated Jonson et al. (1998). The meteorological variability can, to some extent, counteract the gains in the pollution levels achieved through reducing ozone precursor emissions in Europe Hauglustaine et al (2005), Andersson and Engardt (2010). In the presence of volatile organic compounds, the equilibrium favours the formation of higher levels of ozone. Usually the impact of surface ozone on ecosystems and human health is assessed using suitable measures Paoletti et al. (2007), Adams et al (2007). Most of them are proportional to the product of the excess of the concentration over a certain threshold and the exposition time and only one related to the concentration itself is prescribed in the third daughter directive. Its distribution is shown on Figure 11.

**Figure 12:**
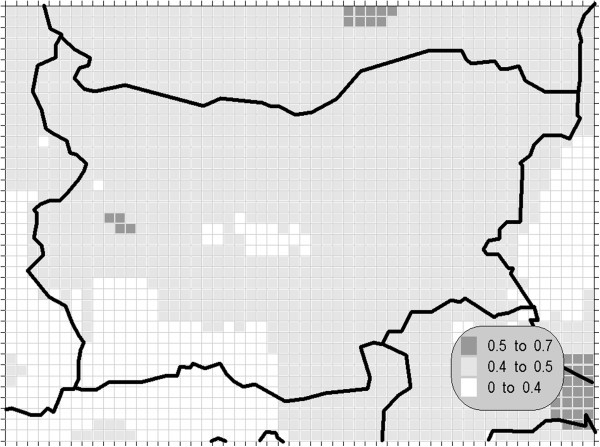
**Maximum of the running 8-hour mean concentration of the carbon monoxide (unit: mg/m**^**3**^**).**

Frequently the carbon monoxide remains outside of the scope of environmental evaluations, similar to this presented here. According to Theakston (2002) the background concentrations of this substance are far below the prescribed limit values and the carbon monoxide air pollution is mostly a problem for indoor microenvironments, in which combustion engines are used under conditions of insufficient ventilation. The fact that carbon monoxide concentrations were regulated by the second daughter directive, however, means that it is on the same level as that of the “classical” pollutants, and is indicative of its relevance for the human health on larger scale. The AQLV for the carbon monoxide, prescribed in the second daughter directive, is only one, and it is the same as for the ozone – the maximum daily 8-hour mean (but the first maximum). Its distribution in the model domain is presented on Figure 12.

**Figure 13:**
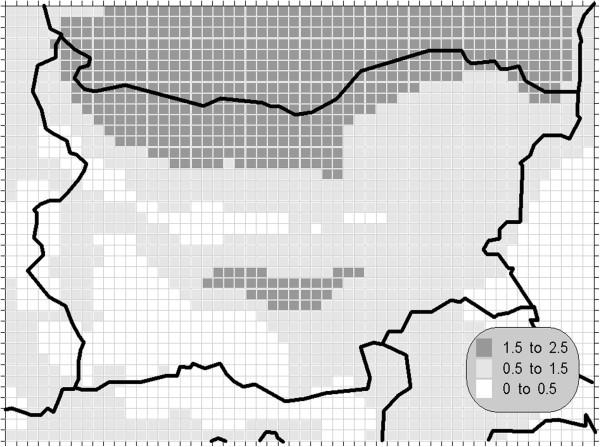
**Annual mean concentration of the ammonia (unit: μg/m**^**3**^**).**

**Figure 14:**
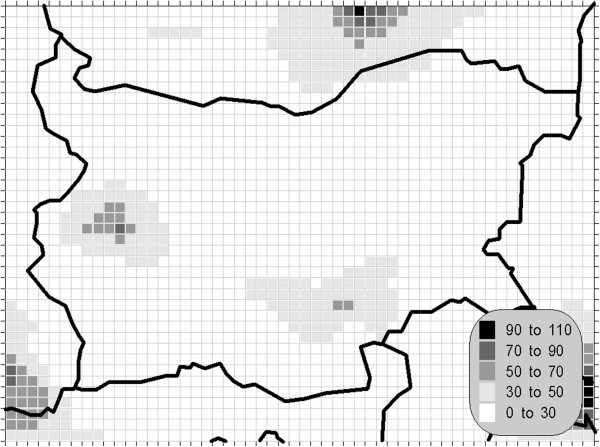
**Maximal hourly concentration of the ammonia (unit: μg/m**^**3**^**).**

Most of earth’s biodiversity is found in natural and seminatural ecosystems, both in aquatic and terrestrial habitats. Man’s activities pose a number of threats to the structure and functioning of these ecosystems, and thus to the natural variety of plant and animal species. One of the major threats in recent years is the increase in airborne nitrogen pollution, which is not limited to the nitrogen oxides only. The ammonia and the ammonia ions are also widespread nitrogen containing atmospheric contaminants with significant environmental significance. Nitrogen is the only nutrient whose cycle through the ecosystem is almost exclusively regulated by biological processes. There have been important developments in the use of critical level and critical load approaches for setting air quality guidelines, but mainly because some remaining gaps in knowledge prescribed AQLV in the current European legislation still are missing. There are insufficient data to provide these levels with confidence at present, but according Theakston (2002) the values 270 μg/m^3^ as 24-hour means and 8 μg/m^3^ as an annual mean are accepted. Figures 13 and 14 show the calculated results for these two statistical quantities.

## Comment of the results and conclusion

One of the most relevant results of the presented study is the confirming of the significant overrun of all AQLV for the sulphur dioxide over a large territory of the model domain. The most polluted areas are in the vicinity of the TPP *Maritsa-Istok* – a set of three coal-burning thermal power plants. The annual mean and winter concentrations there are in the range 20–45 μg/m^3^, with 98.90 percentile (4^th^ daily maximum) in the range 125–225 μg/m^3^. The daily mean AQLV is bridged at least once over significantly larger area. The situation with the shortest-term AQLV, the 99.71 percentile (25^th^ hourly maximum) of the hourly maxima is similar: over the same region this parameter is in the range 350–650 μg/m^3^ and in same grid cells the peak (the first maximum) is over 1000 μg/m^3^. The AQLV for this value is bridged also at least once over very large territory.

The pollution patterns for the NO_x_ statistics are typical of this contaminant –most affected are the areas close to the significant sources – the biggest cities in the domain (Sofia, Bucurest and Istanbul in the lower right corner of the grid) where the road traffic is very intense. The calculated values are, however, relevantly lower then the prescribed AQLV.

The situation of the pollution with the surface ozone causes concern – practically over the whole domain the running 8-hour mean is over the threshold of 120 μg/m^3^ and the 92.88 percentile (26^th^ maximum of this statistics) bridges this limit over large territories – over the Black sea, due mainly to the small deposition over water and over the mountainous southern part of the country. This pattern is similar to the distribution of the ozone exposure indexes, calculated by the Chervenkov (2012) using the same datasets.

With broad maxima around the strongest sources, the distribution of the PM_10_-air pollution is also typical. With peak values below 15 μg/m^3^ the annual average is far from the corresponding AQLV and although that in some regions the maximum 24-hour mean is up to 35 μg/m^3^, the 97.81 percentile of this parameter is everywhere smaller than 50 μg/m^3^, which is the corresponding AQLV. At first sight these results contradict many news reports from some Bulgarian cities of episodes with extremely high aerosol air pollution. Actually, such results are often the consequence of the combination of rare meteorological conditions, such as grounding of plumes from point and/or area sources and sudden, not encountered in the inventory procedure, emissions. In all cases such episodes remain limited spatially over very small territories and the results of this study describes more or less background conditions. The concentration statistics of the remaining pollutants, namely the fine particulate matter, the carbon monoxide and the ammonia, are far below the corresponding AQLV with values comparable to the European background levels, pointed out in Theakston (2002).

The air pollution with sulphur dioxide is evidently a matter of great concern. Obviously despite the modernization, in particular the installation of desulphurization facilities in the last decade of the former century, TPP *Maritsa-Istok* remains one of the most significant source of air pollution in Bulgaria. The significant overrun of AQLV in all time scales over big area is a prerequisite for the whole complex of problems connected with this substance – from the acute pulmonary response in the sensitive part of the population, caused by short-term peak concentrations to deep environmental consequences as acidification and material deterioration (Chervenkov 2007), which are linked with long-lasting concentrations far below those known to be harmful to humans.

The study confirms also generally the worrying picture with the ozone pollution. It is pronounced in regions with strong photochemical activity, such as the Mediterranean basin and Balkan Peninsula. Due to its central location in the second region, Bulgaria may be considered as a hot-spot for ozone and representative of ozone effects on Balkan ecosystems. The bridging of the ozone AQLV like the exposure indexes over almost the whole model domain is disturbing. The general worrying picture of the air pollution with sulphur dioxide and ozone reveals the acute necessity of more detailed studies, based on data from recent years.

For certain compounds, such as the remaining in this study, the proposed health- and environmental related guidelines are orders of magnitude higher than current ambient levels. The fact that existing modelled levels for some substances are much lower than the guideline levels by no means implies that pollutant burdens may be increased up to the guideline values. Any level of air pollution is a matter of concern, and the existence of guideline values never means a license to pollute.

At the end, we need to emphasize that the presented results have to be accepted carefully because such a model evaluation is only an approximation to a set of complex processes. Using another CTM or climate model would give different details, but generally a general agreement with several of other similar works, in particular these of the other participants Zlatev (2009), Huszar et al. (2011) of the CECILIA WP7 can be found. The study has to be treated only as a step toward revealing some aspects of the air pollution over the model domain in the last decade of the former century as an element of a more effective strategy of mitigation and abatement of the negative health and environmental tendencies.
